# Combinatorial macrophage induced innate immunotherapy against Ewing sarcoma: Turning “Two Keys” simultaneously

**DOI:** 10.1186/s13046-024-03093-w

**Published:** 2024-07-11

**Authors:** Wen Luo, Hai Hoang, Katherine E. Miller, Hongwen Zhu, Serena Xu, Xiaokui Mo, Elizabeth A. R. Garfinkle, Heather Costello, Saranga Wijeratne, Wiebke Chemnitz, Ronan Gandhi, Yanling Liao, Janet Ayello, Aliza Gardenswartz, Jeremy M. Rosenblum, Kevin A. Cassady, Elaine R. Mardis, Dean A. Lee, Timothy P. Cripe, Mitchell S. Cairo

**Affiliations:** 1 Department of Pediatrics, New York Medical College, 15 Dana Road, Valhalla, NY 10595 USA; 2 Department of Pathology, Immunology and Microbiology, New York Medical College, Valhalla, NY 10595 USA; 3 Institute for Genomic Medicine, Nationwide Children’s Hospital, Columbus, OH USA; 4 Department of Pediatrics, The Ohio State University, Columbus, OH USA; 5 James J. Peters Veterans Affairs Medical Center, Bronx, NY USA; 6 Department of Biomedical Informatics, Center for Biostatistics, The Ohio State University, Columbus, OH USA; 7 Department of Medicine, New York Medical College, Valhalla, NY USA; 8 University of Chicago, Chicago, IL USA; 9 Center for Childhood Cancer Research, Nationwide Children’s Hospital, Columbus, OH USA; 10 Department of Neurosurgery, The Ohio State University, Columbus, OH USA; 11 Department of Cell Biology and Anatomy, New York Medical College, Valhalla, NY USA

**Keywords:** CD47 blockade, Chemotherapy, Macrophages, Ewing sarcoma

## Abstract

**Background:**

Macrophages play important roles in phagocytosing tumor cells. However, tumors escape macrophage phagocytosis in part through the expression of anti-phagocytic signals, most commonly CD47. In Ewing sarcoma (ES), we found that tumor cells utilize dual mechanisms to evade macrophage clearance by simultaneously over-expressing CD47 and down-regulating cell surface calreticulin (csCRT), the pro-phagocytic signal. Here, we investigate the combination of a CD47 blockade (magrolimab, MAG) to inhibit the anti-phagocytic signal and a chemotherapy regimen (doxorubicin, DOX) to enhance the pro-phagocytic signal to induce macrophage phagocytosis of ES cells in vitro and inhibit tumor growth and metastasis in vivo.

**Methods:**

Macrophages were derived from human peripheral blood monocytes by granulocyte–macrophage colony-stimulating factor (GM-CSF) and macrophage colony-stimulating factor (M-CSF). Flow cytometry- and microscopy-based in-vitro phagocytosis assays were performed to evaluate macrophage phagocytosis of ES cells. Annexin-V assay was performed to evaluate apoptosis. CD47 was knocked out by CRISPR/Cas9 approach. ES cell-based and patient-derived-xenograft (PDX)-based mouse models were utilized to assess the effects of MAG and/or DOX on ES tumor development and animal survival. RNA-Seq combined with CIBERSORTx analysis was utilized to identify changes in tumor cell transcriptome and tumor infiltrating immune cell profiling in MAG and/or DOX treated xenograft tumors.

**Results:**

We found that MAG significantly increased macrophage phagocytosis of ES cells in vitro (*p* < 0.01) and had significant effect on reducing tumor burden (*p* < 0.01) and increasing survival in NSG mouse model (*p* < 0.001). The csCRT level on ES cells was significantly enhanced by DOX in a dose- and time-dependent manner (*p* < 0.01). Importantly, DOX combined with MAG significantly enhanced macrophage phagocytosis of ES cells in vitro (*p* < 0.01) and significantly decreased tumor burden (*p* < 0.01) and lung metastasis (*p* < 0.0001) and extended animal survival in vivo in two different mouse models of ES (*p* < 0.0001). Furthermore, we identified CD38, CD209, CD163 and CD206 as potential markers for ES-phagocytic macrophages. Moreover, we found increased M2 macrophage infiltration and decreased expression of Cd209 in the tumor microenvironment of MAG and DOX combinatorial therapy treated tumors.

**Conclusions:**

By turning “two keys” simultaneously to reactivate macrophage phagocytic activity, our data demonstrated an effective and highly translatable alternative therapeutic approach utilizing innate (tumor associated macrophages) immunotherapy against high-risk metastatic ES.

**Supplementary Information:**

The online version contains supplementary material available at 10.1186/s13046-024-03093-w.

## Introduction

Ewing sarcoma (ES) is a malignant bone and soft tissue tumor. Over the last 40 years, despite multiple therapeutic approaches including surgery, chemotherapy, radiation, megatherapy [1] and small molecule and immune targeted therapy [2–8], metastatic and recurrent/refractory ES has a dismal prognosis (< 25% overall survival) [9]. Novel therapeutic strategies are urgently needed.

Macrophages play critical roles in various physiological functions from phagocytosis to antigen presentation, wound healing, and inflammation among others [10]. Macrophages adopt distinct polarization subtypes in response to different environmental stimuli, spanning a broad spectrum of intermediate subtypes with M1 (classically activated) at one extreme and M2 (alternatively activated) at the other [11]. In a highly immune suppressive tumor microenvironment (TME), tumor associated macrophages (TAM) are often polarized to the M2 subtype and have been implicated in promoting tumorigenesis and dissemination [12]. In ES patient tumors, M2 TAMs are the most abundant immune cells [13], conferring a poor prognosis [14]. However, M2 macrophages are known to harbor similar, if not higher, phagocytic activity than M1 macrophages against tumor cells [15]. Better understanding of how tumor cells evade macrophage phagocytosis will facilitate discovery of novel approaches to enhancing the ability of macrophages in recognizing and destroying tumor cells.

Up-regulation of the “don’t eat me” signal mediated by the CD47/SIRPα (macrophage signal regulatory protein α) axis is such a mechanism being extensively studied [16]. Binding of CD47 to SIRPα results in tyrosine phosphatase activation and inhibition of myosin accumulation at the assembly site of the phagocytic synapse, leading to inhibition of phagocytosis [17]. Tumor cells express high levels of CD47 to enable immune evasion from macrophage clearance for survival and spreading [16]. This signal can be therapeutically targeted by a blocking anti-CD47 antibody, which was demonstrated to be efficacious in certain types of cancer such as Non-Hodgkin lymphoma [18]. However, the efficacy of CD47 blockade as a monotherapy for most cancers is limited [19, 20], suggesting that CD47/SIRPα axis is not the sole mechanism of resistance to macrophage induced phagocytosis of tumor cells.

Indeed, macrophage phagocytosis is also governed by the pro-phagocytic “eat me” signals, typically mediated by cell surface calreticulin (csCRT). CRT is an endoplasmic reticulum associated chaperone protein [21]. In the context of immunogenic cell death, CRT translocates to the cell surface and mediates phagocytosis of dying cells by interacting with low density lipoprotein receptor-related protein 1 (LRP1 or CD91) on phagocytes [22]. CRT levels are found down-regulated in multiple advanced cancers, correlating with reduced major histocompatibility complex I expression and poor patient outcome [23, 24]. This finding suggests that elevating CRT levels on tumor cells may be a therapeutic approach. It should be noted that CRT mediated pro-phagocytic signal can be counterbalanced by overexpression of CD47 as documented in leukemia patient samples [25].

We and others found that ES cells and tumors overexpress CD47 but express no or low levels of csCRT [26]. We hypothesize that simultaneously enhancing the csCRT-mediated “eat me” signal and inhibiting the CD47-mediated “don’t eat me” signal will effectively boost the phagocytic activity of TAMs and result in significantly impaired ES tumor growth and metastasis (Supplemental Fig. 1). Doxorubicin (DOX) is one of the first line chemotherapy drugs utilized in ES patients and is known to enhance translocation of intracellular CRT to the surface of mouse colon cancer cells [27]. Magrolimab (MAG) is an investigational monoclonal antibody against CD47 and is designed to block the “don’t eat me” signal [28]. Here, we aim to investigate whether DOX elicits csCRT expression on ES cells and further enhance MAG induced increase in phagocytic and anti-tumor activities of macrophages against ES.

**Fig. 1:**
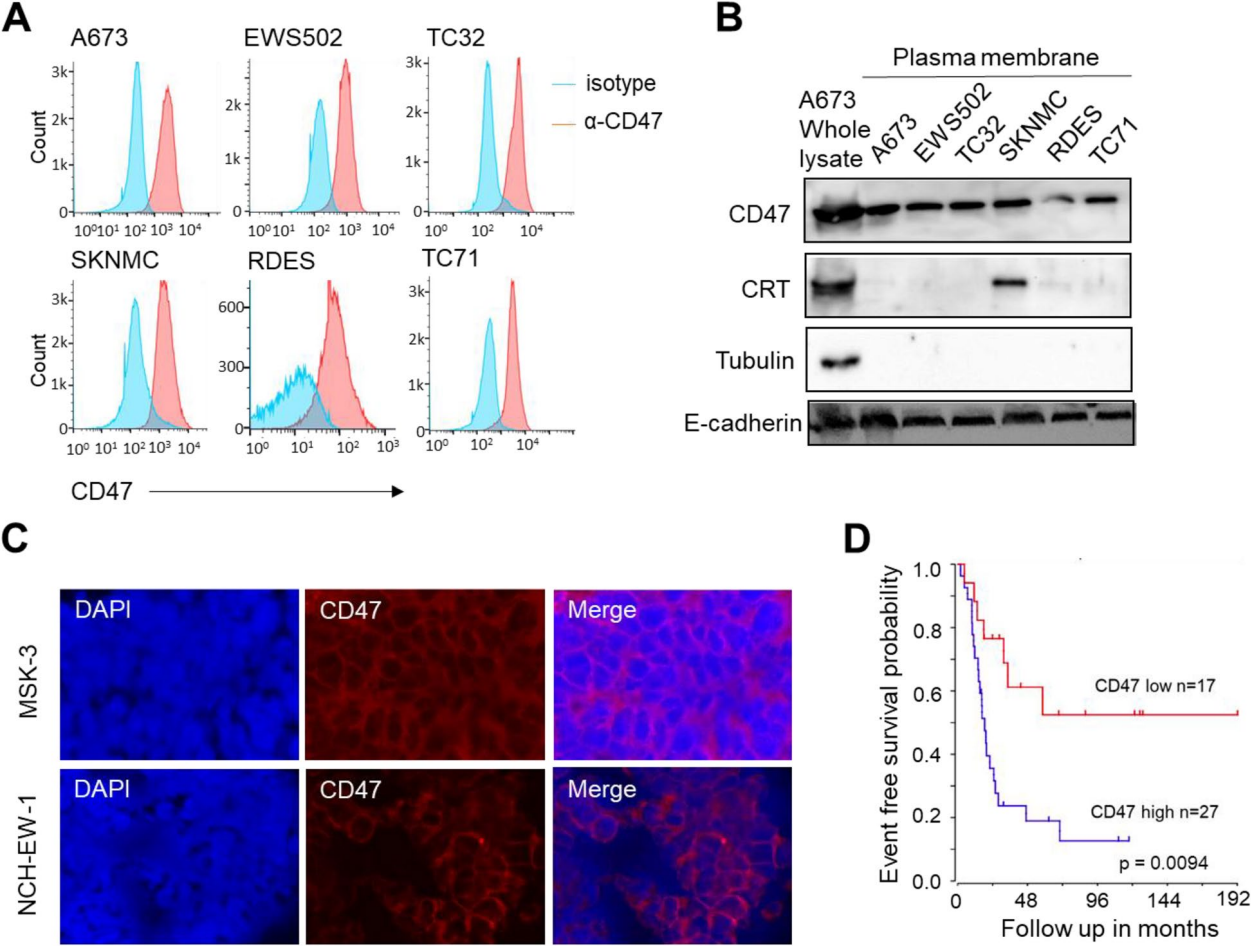
CD47 is overexpressed on ES cells and PDX tumors and its expression level correlates with ES patient outcome. **A** CD47 expression levels detected by flow cytometry on ES A673, EWS502, TC32, SKNMC, RDES, and TC71 cells. **B** CD47 and CRT expression levels on ES cell surface. Plasma membrane proteins were extracted and separated by SDS-PAGE electrophoresis. CD47 and CRT were detected by western blotting analysis. A673 whole cell lysate was used as a positive control sample. Tubulin and E-cadherin were used as loading controls. **C** The expression level of CD47 on ES PDX tumors MSK-3 and NCH-EW-1 detected by immunostaining.** D** Kaplan–Meier survival curves of ES patients from the study of Savola et al. based on CD47 expression levels on the tumors. Patient samples were taken prior to any treatment in 29 cases. Chemotherapy, radiation therapy and/or surgical treatment was applied before material was collected from all the other patients. The patient tumor samples included primary and recurrent tumors and metastases. Detailed clinical information is provided in Supplemental Table 1. Patients whose tumors had high CD47 expression levels (*n* = 27) had a significantly worse event free survival (logrank *P* value = 0.0094) than patients who had low CD47 expressing tumors (*n* = 17)

## Materials and methods

### Cell lines, patient derived xenografts (PDX) and reagents

A673, TC32, RDES and Raji cells were obtained from American Type Culture Collection (ATCC) and grown according to ATCC recommendations. EWS502 was grown in 15% fetal calf serum in RPMI media. SKNMC and TC71 were grown in 10% fetal bovine serum in DMEM and RPMI media, respectively. Cell lines are tested yearly for mycoplasma contamination using a polymerase chain reaction-based detection kit (Southern Biotech) and cells are authenticated by short tandem repeat profiling (Genetica DNA Laboratories). Ewing sarcoma PDXs MSK-3 and NCH-EW-1 were obtained from the Biospecimen Core of Nationwide Children’s Hospital (Columbus, OH). DOX was obtained from THYMOORGAN PHARMAZIE GmbH (Germany). MAG was generously supplied by Gilead Sciences.

### DNA constructs

The clustered regulatory interspaced short palindromic repeats (CRISPR)/Cas9 knockout constructs for CD47 were created by cloning the CRISPR guide RNAs against CD47 (5’-CCTGGTAGCGGCGCTGTTGC-3’ (KO1); 5’-TCCATGCTTTGTTACTAATA-3’ (KO2)) into the lentiCRISPRv2 vector (Addgene plasmid #52961) [29]. Guide sequences were designed using the Broad Institute sgRNA designer tool (https://portals.broadinstitute.org/gpp/public/analysis-tools/sgrna-design).

### Plasma membrane protein purification and detection

The Pierce Cell Surface Protein Isolation Kit (89,881, ThemoFisher Scientific) was used to extract plasma membrane proteins according to the manufacturer’s instructions. Cell surface CRT and CD47 expression was subsequently detected by SDS-PAGE electrophoresis and immunoblotting using anti-CRT antibody (PA3-900, ThermoFisher Scientific) and anti-CD47 antibody (63000S, Cell Signaling Technology), respectively. Tubulin and E-cadherin expression was detected by anti-tubulin (sc-5286, Santa Cruz Biotechnology) and anti-E-cadherin (3195, Cell Signaling Technology) antibodies, respectively, as loading controls.

### Generation of macrophages

Generation of monocyte-derived macrophages was performed as previously reported [30] with minor modifications. Briefly, donor peripheral blood mononuclear cells were isolated by density gradient using Ficoll-Paque PLUS (GE17-1440–03, Millipore sigma) and CD14 + monocytes were purified using microbeads (130–050-201, Miltenyi Biotec). Peripheral blood mononuclear cells (1 × 10^7^ cells/ml) or monocytes (5 × 10^5^ cells/ml) were seeded in RPMI with 10% FBS in 10 cm tissue culture treated plates for 6 days in the presence of either 100 ng/ml recombinant human GM-CSF (130–095-372, Miltenyi Biotec) or M-CSF (130–096-491, Miltenyi Biotec) with replenishment of media containing either growth factor on day 4. We named the induced macrophages GM-CSF-Mφ and M-CSF-Mφ based on the recommendation for macrophage nomenclature by Murray et al. [31].

### Flow cytometry

Cells were stained with antibodies in FACS buffer (Dulbecco's phosphate-buffered saline with 0.5% bovine serum albumin) in the dark at 4℃ for 1 h and washed in FACS buffer after incubation. Antibodies used include α-CD47 (B6H12, ThermoFisher Scientific, 11–0479-42), α-Calreticulin (ThermoFisher Scientific, PA3-900), α-CD11b (Miltenyi Biotec, 130–110-554), α-CD14 (R&D Systems, FAB3832P-025), α-CD38 (R&D Systems, FAB2404P), α-CD68 (BioLegend, 333,819), α-CD80 (BioLegend, 305,220), α-CD163 (R&D Systems, FAB1607P-025), α-CD206 (R&D Systems, FAB25342P), α-CD209 (R&D Systems, FAB161P-025), Alexa Fluor 488 donkey α-rabbit IgG (Invitrogen, A21206).

### Flow-based in-vitro phagocytosis assay

Tumor cells were labeled with CellTracker Green CMFDA dye (C2925, ThermoFisher Scientific) as per manufacturer’s instructions and treated with or without 2–20 µg/mL DOX (Thymoorgan Pharmazie GmbH, Germany) for 4–24 h and washed once with PBS before co-culturing with GM-CSF-Mφ or M-CSF-Mφ at a 2:1 ratio together with or without MAG (Gilead Sciences) at a concentration of 1–10 µg/mL in RPMI for 2–4 h. Cells were harvested and washed with cold PBS and stained with α-CD11b. Flow cytometry was carried out and phagocytosis index was measured as the percentage of CD11b + FITC + macrophages in the total CD11b + macrophages and normalized to the control condition.

### Immunofluorescent-based in-vitro phagocytosis assay

GM-CSF-Mφ and M-CSF-Mφ were stained with CellTracker Blue CMAC dye (C2110, ThermoFisher Scientific) and tumor cells with Green CMFDA dye and co-cultured at a 1:2 ratio together with or without 1 µg/mL MAG in RPMI for 4 h. Where indicated, tumor cells were treated with 10 µg/mL DOX for 16 h before co-culturing with macrophages. Fluorescent images were taken using the EVOS M5000 imaging system (ThermoFisher Scientific).

### Apoptosis assay

Annexin V-FITC apoptosis staining/detection kit (ab 14,085, Abcam) was used to detect early apoptosis according to manufacture instructions. Briefly, cells were treated with or without 2–20 ug/mL of DOX for 4–24 h. Cells were washed twice with cold PBS and resuspended in 1xbinding buffer with Annexin V-FITC and incubated at room temperature for 15 min in the dark before flow cytometry analysis.

### RNA-Seq analysis

Total RNA was extracted from tumors using RNeasy mini kit (74,104, Qiagen) after homogenization of tumors by Tumor Dissociation Kit (130–096-730, Miltenyi Biotec) combined with the gentleMACS Dissociator (Miltenyi Biotec) according to manufacturer instructions. RNA was treated with RNase free DNase (79,254, Qiagen) and Ribo-Zero capture beads (20,037,135, Illumina) to deplete DNA and ribosomal contaminants, respectively. RNA was then used as input for Illumina TruSeq Stranded Total RNA library preparation kit and sequenced on NovaSeq 6000 to obtain ~ 60 M reads per sample. To prevent any bias that would occur from aligning to one genome at a time, RNA-Seq reads were aligned using STAR (STAR_2.6.1c) to a concatenated reference comprised of the Homo sapiens (GRCh38.p12 assembly) and Mus musculus (GRCm38.p6 assembly) genomes. The resulting alignments were separated into species-specific bam files, converted back to fastq files and processed through individual human and mouse pipelines, thus creating individual alignments and transcript counts per genome. DESeq2 (v1.30.1) was used to normalize expression values and to identify differentially expressed genes between groups [32]. A threshold for differentially expressed genes (DEG) between the two groups was set to an absolute value of fold change (FC) ≥ 1.5 and a false discovery rate of ≤ 0.10. Genes passing these thresholds were used for generating volcano plots using the “EnhancedVolcano” package in R version 4.1.1 (https://bioconductor.org/packages/release/bioc/vignettes/EnhancedVolcano/inst/doc/EnhancedVolcano.html). Genes displayed on the plots are colored according to their FC value. Genes with a high FC value (> 2.5) are colored red, genes with a low FC value (< -2.5) are colored blue, and genes with a mid FC value (between 2.5 and -2.5) are colored purple.

### Functional annotation and enrichment analyses

Human and mouse DEG sets were submitted to the Database for Annotation, Visualization and Integrated Discovery (DAVID) (david.abcc.ncifcrf.gov) for enrichment analysis with the Functional Annotation Tool, where OFFICIAL_GENE_SYMBOL was selected and the whole genome of *Homo sapiens* and *Mus musculus* were used as the background genes, respectively.

### CIBERSORTx analysis

CIBERSORTx (https://cibersortx.stanford.edu) was used to predict the composition of mouse immune cell types in the xenograft tumors using the RNA-Seq mouse gene expression normalized values as input mixture file [33] and ImmuCC as the signature file [34]. ImmuCC is a gene signature reference file of 25 mouse immune cell types derived from microarray expression data.

### Animal studies

All animal studies were performed in accordance with protocols approved by the New York Medical College Institutional Animal Care and Use Committee. Luciferase expressing A673 cells or ES PDX tumors (NCH-EW-1) were implanted into the tibia (2 × 10^5^ cells/site) or flanks (1 × 10^6^ cells or a 5 × 5 × 5 mm piece per site) of 4–6 weeks old female/male NOD SCID gamma (NSG) mice. After validation of tumor engraftment by Xenogen IVIS imaging or when PDX tumors reach 200 mm^3^, control (PBS and immunoglobulin G (IgG)) or DOX (0.5 mg/kg, once every 3 days for 5 times, intravenous) or MAG (150 µg/animal, once per day for 12 days, intraperitoneal) or DOX combined with MAG were injected. No randomization or blinding was used. Tumor growth was monitored by caliper measurement daily and IVIS imaging weekly [5]. Mice were followed until death or sacrificed upon reaching a tumor size of 2 cm in any dimension when the tumors and/or lungs were harvested.

### Survival analysis for microarray data

To analyze the microarray data (accession number GSE17679), patients were dichotomized into CD47 high and low groups based on median expression level. R2 Genomics Analysis and Visualization Platform (https://r2.amc.nl/, Amsterdam UMC) was used to plot the Kaplan Meier survival curves to show event free survival probability of ES patients (Mixed Ewing sarcoma – Savola – 117 dataset) [35] with high versus low CD47 expressing tumors. Log rank method was used to test the difference between the two curves. Detailed clinical information is provided in the online Supplemental Table 1.

### Statistical analyses

Analysis of variance (ANOVA) was used to analyze experiments with multiple independent groups. In-vivo tumor growth and the interaction between two reagents in terms of growth rate was analyzed by mixed effect model, accounting for observational dependencies for each subject. The Fisher’s Exact test was used to compare the percentages of mice with lung metastasis between treatments. For mouse survival experiments, Kaplan Meier method was used to display survival probabilities, and compared by log-rank tests. Additionally, the interaction/synergy of the two reagents was tested using the Cox regression model. ANOVA, mixed effect modeling and Cox regression were conducted using SAS 9.4 (SAS Institute, Cary, NC), and Fisher’s Exact test was performed in GraphPad StatMate version 2.00. Before conducting mouse experiments, sample sizes achieving 80% power to detect an effect size > 2 were determined at significant level as 0.05 using PASS 20 (Power Analysis and Sample Size Software. NCSS, LLC.). All data are presented as the mean ± SD of at least three independent experiments except where stated.

## Results

### CD47 is highly expressed on ES cells and correlates with poor patient outcomes

To determine whether CD47 is a potential target in ES, we evaluated CD47 expression levels on multiple ES cell lines (A673, EWS502, TC32, SKNMC, RDES and TC71). Both flow cytometry analysis of the cells and western blotting of purified cell surface proteins showed high levels of CD47 on the surface of ES cells (Fig. 1A and B). In addition, CD47 expression was readily detected on ES patient-derived xenografts (MSK-3 and NCH-EW-1) by immunofluorescent analysis (Fig. 1C). Importantly, via Kaplan–Meier method, we found that higher CD47 expression was associated with worse event free survival in ES patients (logrank *p* value = 0.0094) (Fig. 1D).

### CD47 blockade significantly increased macrophage phagocytosis of ES cells in vitro

**Fig. 2:**
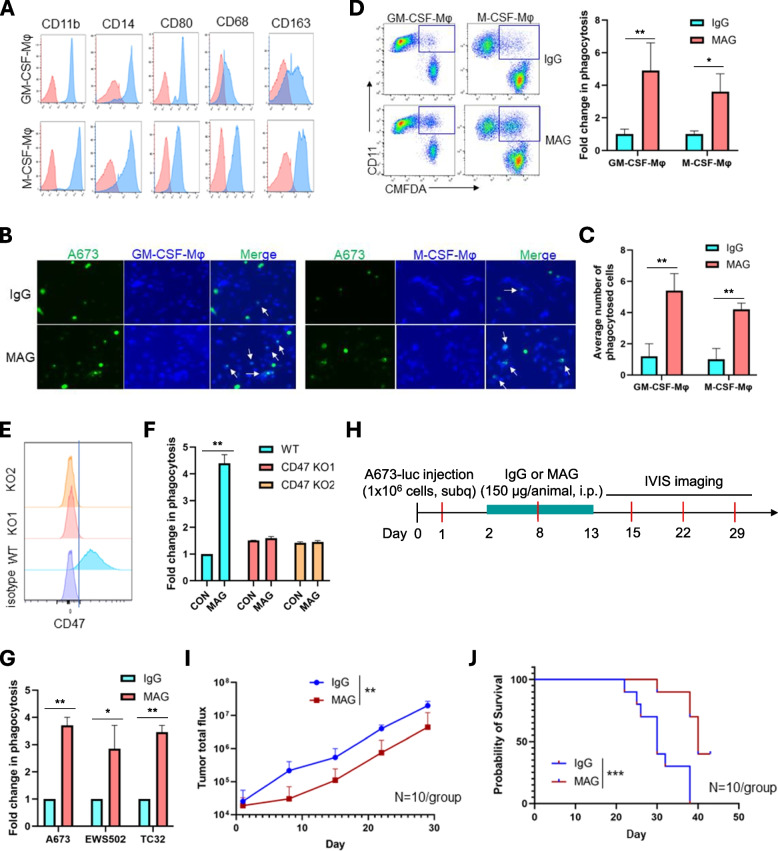
CD47 blockade magrolimab (MAG) enhanced macrophage phagocytosis of ES cells in vitro and had limited anti-tumor effect in vivo against ES. **A** Phenotypes of macrophages derived from peripheral blood monocytes by GM-CSF or M-CSF induction. The peaks in pink represent cells stained with isotypes and the ones in blue are cells stained with antibodies against the indicated surface proteins (CD11b, CD14, CD80, CD68, CD163). **B** Immunofluorescent-based phagocytosis assays showing the increased phagocytosis of A673 cells by both GM-CSF-Mφ and M-CSF-Mφ upon MAG treatment compared to IgG control. A673 cells were labeled with the CMFDA green cell tracker and macrophages the CMAC blue cell tracker. The white arrows are pointing to the macrophages phagocytosed A673 cells. **C** Quantification of the number of macrophages phagocytosed tumor cells in B. Average number of cells in five fields is shown. Error bars represent the standard deviation (STD). **D** MAG treatment enhanced phagocytosis of A673 cells by GM-CSF-Mφ or M-CSF-Mφ compared to IgG control in flow cytometry-based phagocytosis assays. A673 cells were labeled with CMFDA which can be detected by FITC channel. Macrophages were stained with CD11b antibody. The gated FITC + CD11b + cells are macrophages phagocytosed A673 cells. The bar graph shows quantification of the results in three independent biological replicates. **E** Flow cytometry analysis showing CD47 knockout (KO) by the CRISPR/Cas9 approach. **F** Flow cytometry results showing MAG treatment failed to increase macrophage phagocytosis of CD47 KO A673 cells. Quantification of the results in three biological repeat experiments is shown. **p < 0.01; ns, not significant (two-tailed Student t-test). **G** MAG treatment significantly enhanced in-vitro phagocytosis of ES A673, EWS502 and TC32 cells. *p < 0.05, **p < 0.01 (two-tailed Student t-test). **H** Schematic representation of the animal work schedule. Luciferase expressing A673 cells were injected subcutaneously into the flanks of NSG mice. Tumor bearing mice were divided into two groups and treated with IgG and MAG (150 μg/animal, once per day for 12 days, intraperitoneal), respectively. Tumor growth was monitored by weekly IVIS imaging. **I** Tumor growth curves showing significant effects of MAG on primary tumor growth. *N* = 10 per group. **p < 0.01 (ANOVA). **J** Kaplan–Meier curves for comparison of survival between IgG and MAG groups. Animal survival was followed after therapy initiation using death or sacrifice as the terminal event. IgG vs MAG ***p < 0.001 (log rank test)

We derived M1- and M2-like macrophages (GM-CSF-Mφ and M-CSF-Mφ [31]) using GM-CSF and M-CSF respectively from human peripheral blood monocytes (Fig. 2A) and investigated in-vitro phagocytosis of ES cells by both types of macrophages using an immunofluorescent-based phagocytosis assay with or without the CD47 blockade, MAG. We found that in the MAG treated condition, significantly more A673 ES cells were phagocytosed by either GM-CSF-Mφ or M-CSF-Mφ compared to the IgG treated condition (Fig. 2B and C, **p < 0.01). Utilizing a flow cytometry-based phagocytosis assay, we confirmed that MAG resulted in an increased phagocytosis of A673 cells by both types of macrophages (Fig. 2D, **p < 0.01 and *p < 0.05). Given that MAG treatment enhanced phagocytosis of ES cells by both GM-CSF-Mφ (M1-like) and M-CSF-Mφ (M2-like), and the M2-type macrophages are the predominant immune cells in the ES TME [14], we used M-CSF-Mφ in the subsequent phagocytosis assays. To investigate whether the increase in phagocytosis is CD47 specific, we knocked out CD47 in A673 cells (Fig. 2E). CD47 knockout (KO1 and KO2) abolished the effect of MAG on macrophage phagocytosis, demonstrating the effect of MAG is through CD47 (Fig. 2F). We extended the in-vitro phagocytosis assays to three lines of ES cells (A673, EWS502 and TC32) and found that MAG had the same effect on significantly increasing M-CSF-Mφ phagocytosis of these ES cells (*p < 0.05 and **p < 0.01) (Fig. 2G).

### MAG alone had limited effects on reducing tumor growth and prolonging animal survival

To test the efficacy of MAG in vivo, we used an ES xenograft model in NSG mice (Fig. 2H) in which professional phagocytes are present and the binding affinity between human CD47 and murine Sirpα is comparable to that with human SIRPα [36]. We found that MAG treatment significantly decreased tumor growth (**p < 0.01) (F ig. 2I) and resulted in significantly prolonged animal survival compared to the IgG control (***p < 0.001) (Fig. 2J). However, the tumors soon developed resistance, and the long-term animal survival was not improved (Fig. 2J), suggesting MAG alone is not sufficient for long term tumor control.

### DOX elicited cell surface CRT level

**Fig. 3:**
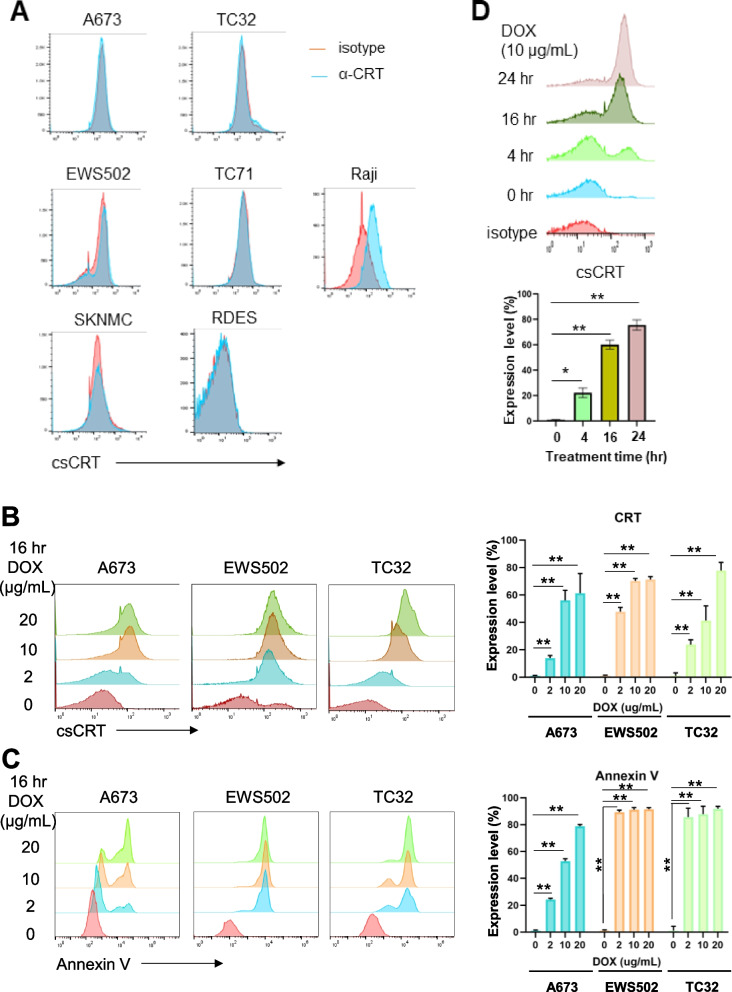
ES cells have no or low CRT expression on the cell surface (csCRT) which can be increased by Doxorubicin (DOX) treatment. **A** Flow cytometry analyses showing csCRT levels on ES A673, EWS502, TC32, SKNMC, RDES and TC71 cells. Raji cells served as a positive control. **B **Representative flow cytometry results showing the csCRT levels on ES cells (A673, EWS502, and TC32) were increased by DOX treatment in a dose dependent manner (*left*). The bar graph shows quantification of the results in three independent biological replicates (*Right*). **p < 0.01 (two-tailed Student t-test). **C **The level of apoptosis in ES A673, EWS502 and TC32 cells was increased by DOX treatment in a dose dependent manner (*left*). The bar graph shows quantification of the results in three independent biological replicates (*Right*). **p < 0.01 (two-tailed Student t-test). ES cells were treated with increasing concentrations (0, 2, 10, 20 µg/mL) of DOX for 16 h before harvested for flow cytometry analyses and evaluation of Annexin V level for apoptosis. **D **Representative result of time course of increase in csCRT levels on ES A673 cells after DOX treatment (*upper*). A673 cells were treated with 10 µg/mL of DOX for different periods of time (0, 4, 16, 24 h) before harvested for flow cytometry analyses. Quantification of the results in three biological repeat experiments is shown in *lower* panel. *p < 0.05, **p < 0.01 (two-tailed Student t-test)

CRT was previously found to be focally expressed in a minor subset (1 in 10) of ES tumor samples in an immunohistochemical analysis [26], suggesting low CRT expression on ES. Indeed, by flow cytometry and immunoblotting of plasma membrane proteins, we found that most of the ES cell lines express minimum levels of CRT on their surface (Figs. 1B and 3A). DOX has been previously demonstrated to enhance csCRT levels during the process of apoptosis in other malignancies [27]. To investigate whether DOX treatment increases csCRT levels in ES, we treated A673, EWS502 and TC32 cells with various concentrations of DOX (0, 2, 10, 20 ug/mL). We found that DOX induced a significant increase in csCRT levels in a dose-dependent manner in all three cell lines (**p < 0.01) (Fig. 3B). This increase in csCRT level correlated with the increase in apoptosis as evidenced by the Annexin V levels (Fig. 3C). We then evaluated csCRT levels after 10 ug/mL of DOX treatment for different periods of time (4–24 h) and found that the csCRT level reached the peak 24 h after DOX treatment (Fig. 3D).

### DOX further enhanced MAG induced macrophage phagocytosis of ES cells in vitro

**Fig. 4:**
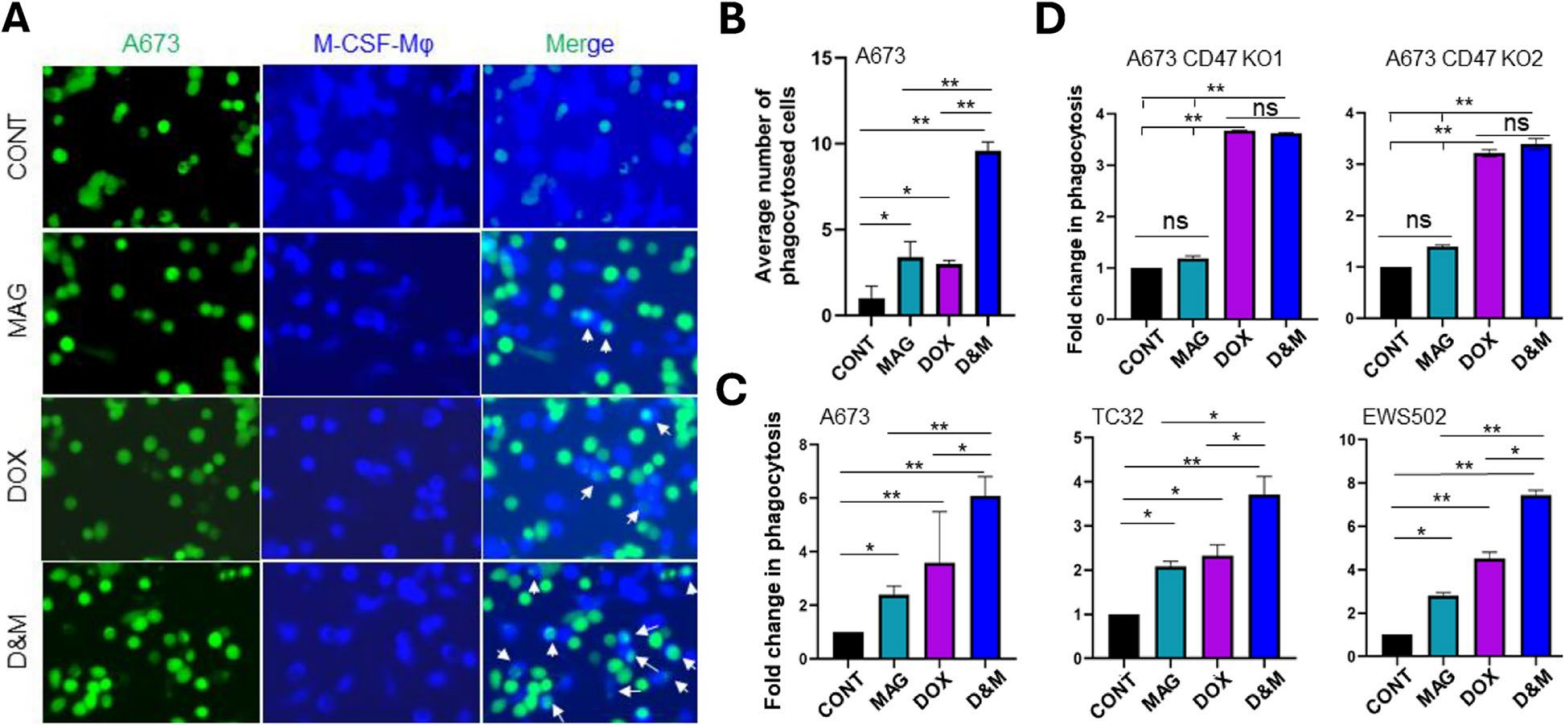
MAG combined with DOX further enhanced macrophage phagocytosis of ES cells. **A** Immunofluorescent-based phagocytosis assays showing representative phagocytosis of A673 cells treated with control or MAG or DOX or MAG combined with DOX (D&M) by M-CSF-Mφ. A673 cells were labeled with the CMFDA green cell tracker and macrophages the CMAC blue cell tracker. The white arrows are pointing to the macrophages phagocytosed A673 cells. **B** Quantification of the number of macrophages phagocytosed tumor cells in A. Average number of cells in five fields is shown. Error bars represent the STD. *p < 0.05, **p < 0.01 (two-tailed Student t-test). **C** Flow cytometry-based phagocytosis assays showing significantly enhanced phagocytosis of ES cells (A673, TC32, EWS502) by MAG alone or DOX alone compared to control which is further enhanced by D&M treatment. Quantification of the results in three biological repeats is shown. *p < 0.05, **p < 0.01 (two-tailed Student t-test). **D** Changes in macrophage phagocytosis of A673 CD47 KO cells after control or MAG alone or DOX alone or D&M treatment. Quantification of the results in three biological repeats is shown. *p < 0.05, **p < 0.01 (two-tailed Student t-test)

To investigate whether DOX-elicited csCRT expression have any effect on CD47 blockade induced enhancement of macrophage phagocytosis of ES cells, we incubated M-CSF-Mφ with ES cells treated with or without DOX in the absence or presence of MAG and performed the immunofluorescent-based phagocytosis assay. We found that the number of phagocytosed A673 cells was significantly increased by either DOX or MAG treatment compared to the control condition (PBS + IgG). Importantly, the combination of the two (D&M) further enhanced macrophage phagocytosis of A673 cells (Fig. 4A and B). Consistently, in the flow cytometry-based phagocytosis assay, we observed the same effect of MAG and DOX on macrophage phagocytosis of three different lines of ES cells (Fig. 4C) (*p < 0.05 and **p < 0.01). Furthermore, CD47 is indispensable for this combinatorial effect, because MAG did not enhance DOX induced macrophage phagocytosis of CD47 KO A673 cells (Fig. 4D).

### Characterization of markers for ES-phagocytic macrophages

**Fig. 5:**
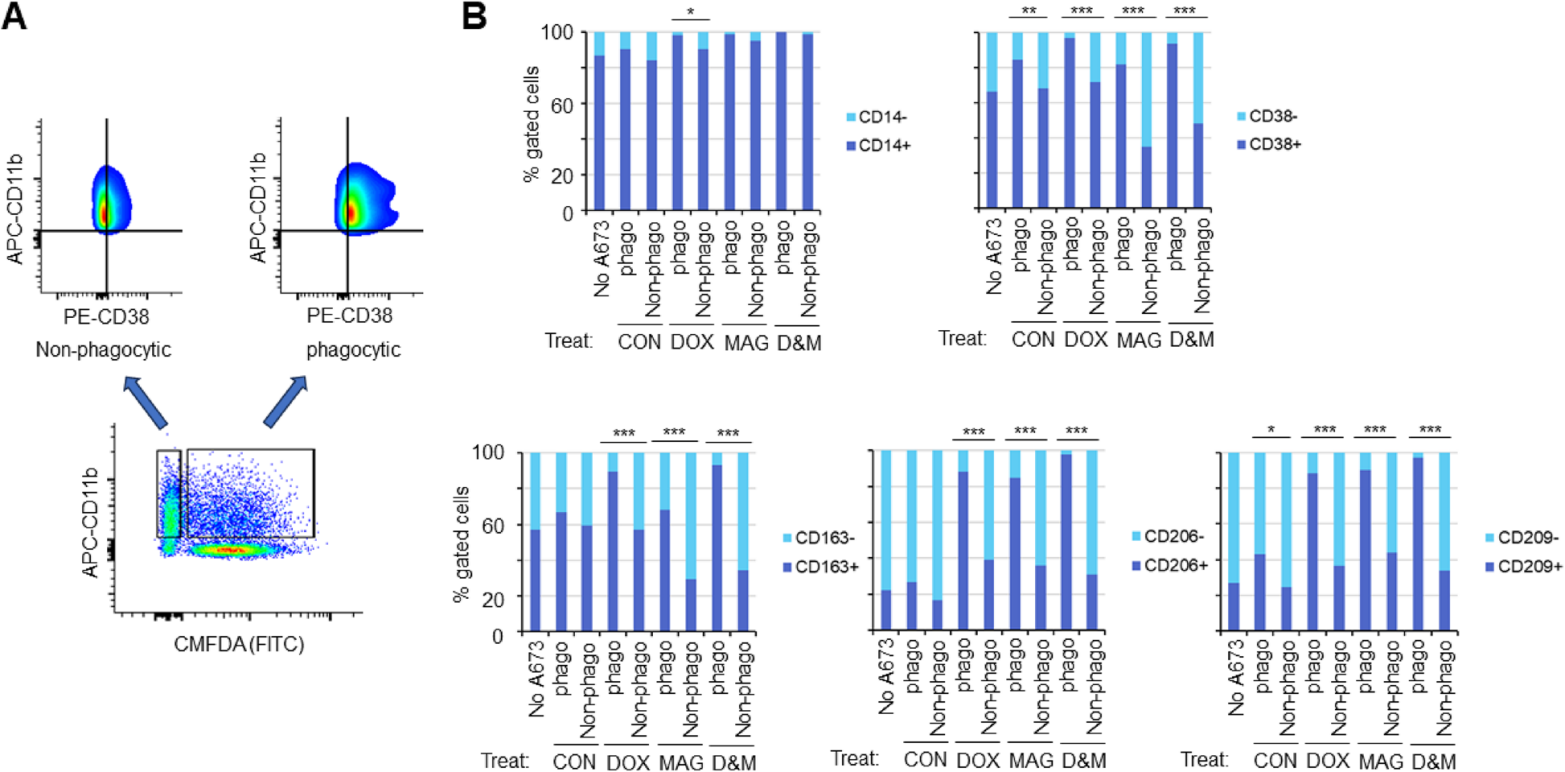
Characterization of markers for ES-phagocytic macrophages. **A** Gating strategy for evaluating cell surface protein expression on phagocytic versus non-phagocytic macrophages. In-vitro phagocytosis assay was carried out incubating A673 cells (Non- or DOX- or MAG- or D&M-treated) labeled with CMFDA (detected by FITC channel) and M-CSF Mφ. Cells were harvested and stained with APC-CD11b and PE-CD38 antibodies followed by flow cytometry analysis. The viable cells were plotted in FITC (CMFDA) x APC view to visualize cell populations in the in-vitro phagocytosis assay. A gate was drawn to include cells that were FITC positive and APC positive as the phagocytic macrophage population, and another gate was drawn to include cells that were FITC- APC + as the non-phagocytic macrophage population. The FITC + APC + and FITC- APC + cells were then plotted separately in a PE x APC view to visualize expression of CD38 in both cell populations. A quat gate was drawn to visualize APC + PE + as the CD38 + and APC + PE- as the CD38- macrophages. **B** Comparison of percent of CD14, CD38, CD163, CD206 or CD209 positive and negative cells in the phagocytic and non-phagocytic macrophages incubated with non- or DOX- or MAG- or D&M-treated A673 cells. Macrophages alone (no A673) served as a control. *p < 0.05, **p < 0.01, ***p < 0.001 (Fisher’s Exact test). Shown are results in a representative experiment. The same trend was seen in three independent biological repeats

Macrophages have been traditionally classified as M1- and M2-type which is a general simplification of the complicated phenotypes of macrophages. Currently, the phenotype of tumor cell phagocytic macrophages is largely unknown. To identify the markers of ES cell phagocytic macrophages, we performed flow cytometry-based phagocytosis assays in the condition of either non- or DOX- or MAG- or D&M treated A673 cells. We plotted the phagocytic macrophages (CD11b + CMFDA (FITC) +) and the non-phagocytic ones (CD11b + FITC-) for expression of various macrophage surface proteins (Fig. 5A). We found that in all four conditions (Non-, DOX-, MAG- or D&M-treated), a significantly higher percentage of CD38 + or CD209 + cells were present in the phagocytic than the non-phagocytic macrophages (Fig. 5B, ***p < 0.001, **p < 0.01 and *p < 0.05). In addition, in DOX- or MAG- or D&M-treated conditions, we found that a significantly higher percentage of CD163 + or CD206 + macrophages were present in the phagocytic than the non-phagocytic macrophage populations (***p < 0.001) (Fig. 5B). These results suggest that CD38, CD209, CD163 and CD206 are potential markers for ES-phagocytic macrophages.

### MAG combined with DOX further significantly limited ES tumor growth and extended animal survival in vivo

**Fig. 6:**
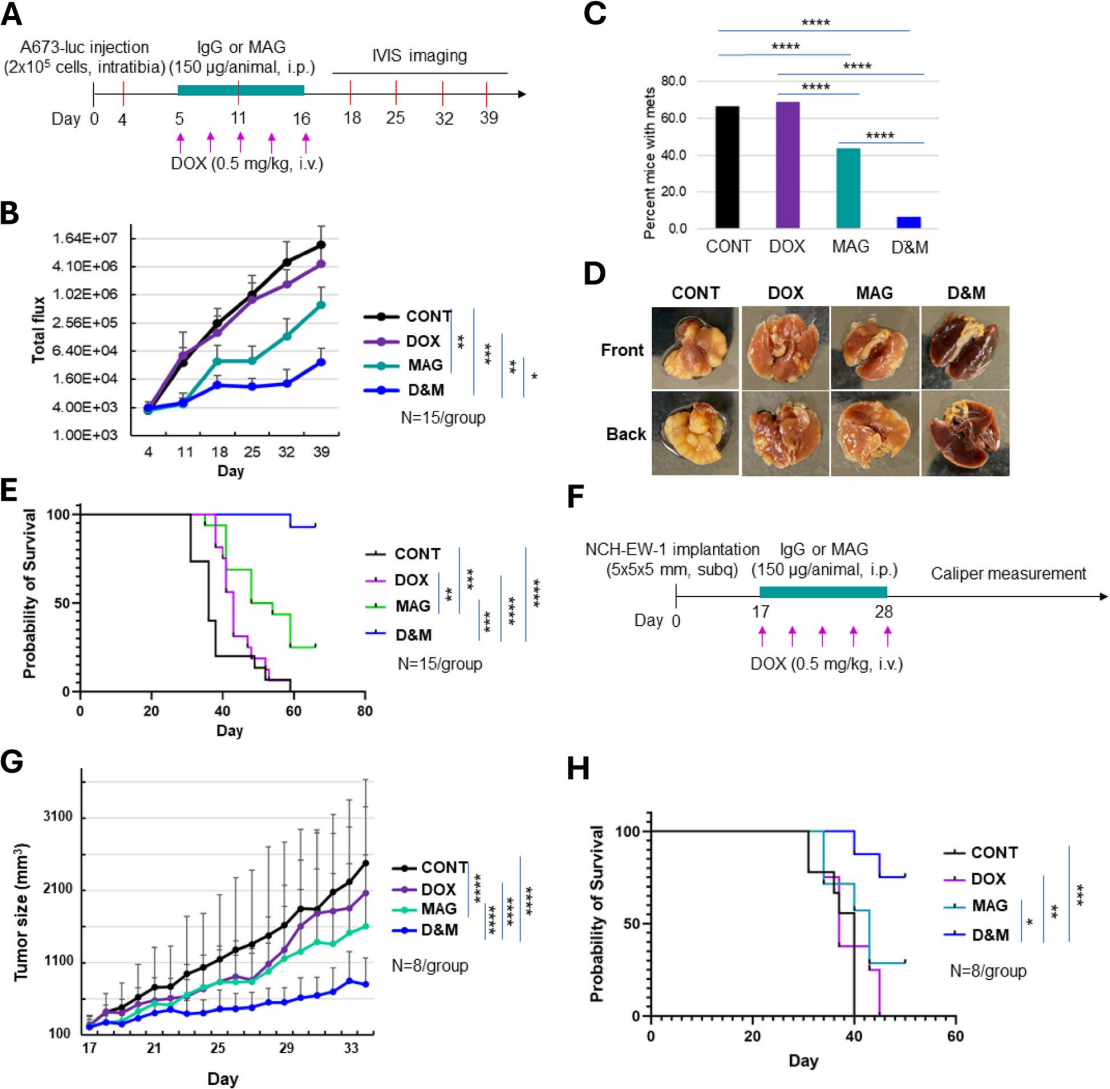
nti-tumor effects of MAG and/or DOX in vivo in a cell-based and a PDX-based xenograft mouse model of ES. **A**, Schematic representation of the animal work schedule. NSG mice with ES xenograft tumors implanted in the tibia were divided into control (PBS + IgG), MAG alone (150 μg/animal), DOX alone (0.5 mg/kg), and D&M treatment groups. *N* = 15 for each group. Tumor growth was monitored by IVIS imaging once a week. **B** Growth curves of primary xenograft tumors in the animals treated with control or MAG or DOX or D&M. *p < 0.05, **p < 0.01, ***p < 0.0001 (ANOVA). Growth rates between groups were analyzed using mixed effect model. The *p*-value of the interaction between MAG and DOX in terms of growth rate is 0.005, indicating a synergy between the two drugs. **C** Graphs showing the percentage of mice with pulmonary lesions in animals treated with control or MAG alone or DOX alone or D&M. ****Fisher’s Exact test *p* < 0.0001. **D** Representative images of lungs from the animals treated with control, DOX, MAG, or D&M. **E** D&M treatment significantly prolonged animal survival compared to control, MAG or DOX treated groups. Mice were followed until death or sacrificed if tumor size reached 2 cm in any dimension. Probability of survival was determined by the Kaplan Meier method using animal death/sacrifice as the terminal event using the Prism program V.8.0 (GraphPad Software). **p < 0.01, ***p < 0.001, ****p < 0.0001 (log rank test). **F** Schematic representation of the animal work schedule in the PDX model. **G** Growth curves of ES PDX tumors in the animals showing synergistic effect of MAG and DOX on decreasing tumor growth (*lower*). *p < 0.05, **p < 0.01, ***p < 0.001, ****p < 0.0001 (ANOVA). NSG mice were implanted with NCH-EW-1 PDX tumors in the flanks. After tumors reach the size of 200 mm.^3^, mice were divided into control (PBS + IgG), MAG alone (150 μg/animal), DOX alone (0.5 mg/kg), and D&M treatment groups. *N* = 8 for each group. Tumor growth was monitored by daily caliper measurement. Growth rates between groups were analyzed using mixed effect model. The *p*-value of the interaction between MAG and DOX in terms of growth rate is 0.0001, indicating a synergy between the two drugs. **H** D&M treatment significantly prolonged animal survival in PDX mouse model compared to control, MAG or DOX treated groups. *p < 0.05, **p < 0.01, ***p < 0.001 (log rank test). Mice were followed until death or sacrificed if tumor size reached 2 cm in any dimension. Probability of survival was determined by the Kaplan Meier method using animal death/sacrifice as the terminal event using the Prism program V.8.0 (GraphPad Software)

We next evaluated the efficacy of MAG or DOX alone and in combination (D&M) in controlling ES xenograft tumor growth using a cell line (A673) based orthotopic mouse model of ES. We found that DOX alone had a minimal, while MAG alone had a moderate effect on xenograft tumor growth. Importantly, MAG combined with DOX (D&M) further significantly decreased ES tumor growth (*p* < 0.01 compared to DOX and *p* < 0.05 compared to MAG alone, the *p* value of the interaction between MAG and DOX in terms of growth rate is 0.005 indicating a synergy between the two drugs) (Figs. 6A and B). Furthermore, MAG treatment significantly reduced the percentage of animals with lung metastatic lesions compared to control or DOX treated animals (42% vs 66% or 68%) and D&M further significantly reduced lung metastasis (5%) (Fig. 6C and D). The superior efficacy of D&M in limiting tumor growth and metastasis gave the animals an advantage of significantly prolonged survival compared to control or either of the single agent treatment (*p* < 0.0001 and *p* < 0.001) (Fig. 6E). Synergy test using Cox model showed the interaction between DOX and MAG is significant (*p* = 0.0335). Consistently, when we extended the in vivo study utilizing a PDX (NCH-EW-1) based ES mouse model, we observed a similar effect of MAG on reducing PDX tumor growth (*p* < 0.0001 compared to control) and that the combination of DOX and MAG (D&M) further significantly reduced tumor growth (*p* < 0.0001 compared to control or either DOX or MAG, the *p* value of the interaction between MAG and DOX in terms of growth rate is 0.0001 indicating a synergy between the two drugs) (Fig. 6F and G). Furthermore, the animal survival was significantly prolonged in the D&M group compared to control (*p* < 0.001) or either of the single agent treated groups (*p* < 0.01, < 0.05 compared to DOX and MAG, respectively) (Fig. 6H).

### MAG increased macrophage infiltration in ES xenograft tumors

**Fig. 7:**
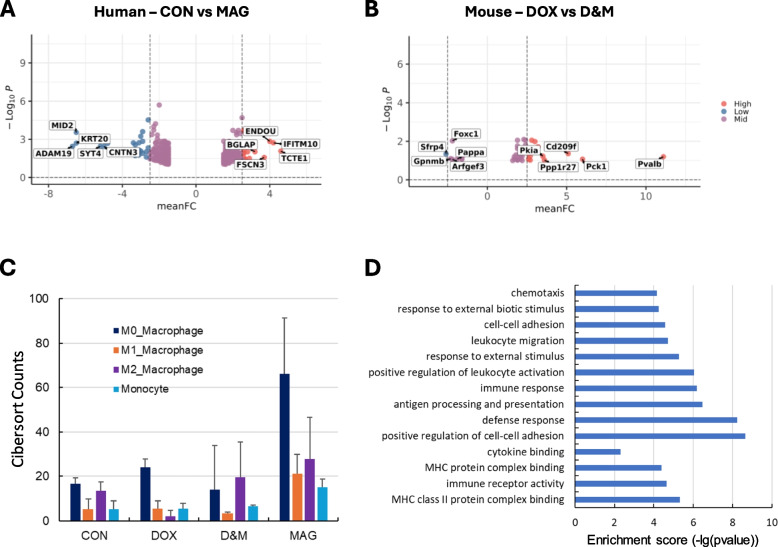
Identification of transcriptomic and immune microenvironmental changes in the MAG and/or DOX treated xenograft tumors. **A** Volcano plot showing significant human DEGs comparing CON and MAG treated tumors. Total RNA was extracted from tumors and subjected to RNAseq analysis. DEGs between the CON and MAG groups were set to a |fold change (FC)|≥ 1.5 and a false discovery rate of ≤ 0.10. Genes passing these thresholds were used for generating volcano plots using “EnhancedVolcano” in R. In the plot, High means FC greater than 2.5, Low means FC less than -2.5 and Mid means FC in between 2.5 and -2.5. **B** Volcano plot showing significant mouse DEGs comparing DOX and D&M treated tumors. RNAseq analysis and generation of volcano plots are as described in A. **C** Changes in the abundance of tumor infiltrating macrophages and monocytes in xenograft tumors. ES xenograft tumors treated with CON or DOX or MAG or D&M were harvested at the end point. Total RNAs were extracted and subjected to RNA-Seq analysis. CIBERSORTx deconvolution of the abundance of mouse immune cell types using RNA-seq gene expression data was carried out using ImmunCC, a signature gene expression reference for 25 mouse immune cell types. Counts for macrophages and monocytes in CON, DOX, MAG, and D&M treated tumors were plotted. **D** Pathway and gene ontology enrichment analysis of the differentially expressed mouse genes comparing DOX and D&M treated tumors by DAVID analysis. Significantly enriched categories were plotted using the enrichment score (-lg (*p* value))

Although the combination of DOX and MAG (D&M) significantly reduced ES xenograft tumor growth, several small tumors developed at the end of the study. We extracted RNA from the tumors in each treatment group and performed RNA-Seq. We compared the transcriptome profiles of the tumors from different groups and identified differentially expressed human genes between groups (Fig. 7A, Supplemental Fig. 2A-D, Supplemental file 1). Via DAVID functional annotation analysis, we found that these differentially expressed genes mostly encode proteins in the plasma membrane and extracellular matrix such as lipid transporters, glycoproteins and collagens (Supplemental Fig. 3A-E). Since the TME is composed of mouse stromal and immune cells, we identified differentially expressed mouse genes between groups (Fig. 7B, Supplemental Fig. 2E-G, Supplemental file 2) followed by pathway and gene ontology enrichment analyses via DAVID. We found that plasma membrane region, cell–cell signaling, and ion channel activity are among the top enriched terms (Supplemental Fig. 3F-J). These data suggest that MAG and/or DOX may regulate cell–cell or protein–protein interaction in the extracellular matrix and/or chemical/signal transduction between the tumor cells and mouse stromal/immune cells in the TME.

To identify the difference in mouse immune cell composition in the TME between different groups, we performed immune cell deconvolution via CIBERSORTx. A drastic increase in the number of macrophages and monocytes in the TME was observed in the MAG treated tumor samples compared to samples from other groups (Fig. 7C). We also noted a moderate increase in the number of M2 macrophages in the D&M treated tumor samples compared to control and DOX alone treated tumors (Fig. 7C). Consistently, we found that chemotaxis, leukocyte migration, response to external stimuli were enriched terms in the gene ontology analysis using differentially expressed mouse genes between DOX and D&M treated tumors (Fig. 7D). These data suggest that MAG alone and combined with DOX increased macrophage infiltration into the ES xenograft tumors.

We next examined more closely the individual top differentially expressed mouse genes between DOX and D&M treated tumors. Among these genes, we found that genes encoding cytokines and proteins that promote M1 macrophage polarization or inhibit M2 macrophage activation including Pck1 and Pvalb were significantly downregulated in D&M treated samples [37–41] (Fig. 7B and Supplemental file 2). Genes that regulate macrophage recruitment and promote M2 macrophage polarization such as Foxc1 and Gpnmb [42, 43] were upregulated in D&M treated samples (Fig. 7B). Interestingly, we found that Cd209f and Cd209g which are the mouse homologs of human CD209 were downregulated in D&M treated samples (Fig. 7B and Supplemental Fig. 2E).

## Discussion

TAMs are abundant in ES patient tumors [14]. Macrophages, including both M1 and M2 subtypes, are known to harbor phagocytic activity against cancer cells [15], yet they fail to phagocytose ES cells and in fact confer a poor prognosis [14] by potentially contributing to ES tumor cell survival and dissemination. In the current study, to overcome the mechanisms underlying the resistance to TAMs associated phagocytosis and convert the “pro-tumor” TAMs into “anti-tumor” phagocytes, we simultaneously blocked the “don’t eat me” signal mediated by CD47 using MAG and boosted the “eat me” signal mediated by cell surface calreticulin (csCRT) using DOX. We demonstrated that the combination of MAG with DOX further significantly increased macrophage phagocytosis of ES cells in vitro (Fig. 4) and significantly reduced tumor growth and lung metastasis of ES xenograft tumors and prolonged animal survival in vivo (Fig. 6). DOX is one of the first line chemotherapy agents utilized in ES patients in the clinic. The combination of DOX with MAG could potentially quickly be translated into a clinical trial for patients with poor risk ES. Furthermore, we characterized the phenotype of phagocytic macrophages in the context of MAG and DOX treated ES cells and for the first time identified CD38, CD209, CD163 and CD206 as the markers for ES-phagocytic macrophages (Fig. 5). Moreover, we identified increased M2 macrophage infiltration and downregulation of phagocytic macrophage marker CD209 in the TME of the MAG and DOX combinatorial therapy treated ES xenograft tumors (Fig. 7).

CD47 is broadly overexpressed across cancer types and represents a potentially widely applicable target for therapeutic blockade in cancer patients. To date, 48 clinical trials on CD47 targeted therapy in various types of cancer have been registered in the USA (clinicaltrials.gov). However, accumulating data have shown that CD47 blockade as a single modality has no or low anti-tumor activity in solid tumors [19, 44]. Our data also showed that MAG alone had significant but short term efficacy in controlling ES tumor growth (Figs. 2 and 6). Macrophage phagocytosis requires both disruption of CD47 “don’t eat me” signals and simultaneous activation of pro-phagocytic “eat me” signals. The “eat me” signals, most often csCRT, are already highly expressed on certain tumors and can be further augmented by tumor specific antigen-targeting antibodies such as rituximab and dinutuximab [44, 45], which bind tumor cells on one end and engage Fc receptors on phagocytes on the other end. However, we and others have found that ES tumors have minimal csCRT expression [26] (Figs. 1 and 3). Thus, effective phagocytosis of ES cells requires enhancing the “eat me” signals.

CRT is an ER (endoplasmic reticulum) protein that translocate to the cell surface when cells are under cellular stress such as chemotherapy. Indeed, when we utilized DOX to treat ES cells, we observed an increase in csCRT levels on ES cells (Fig. 3). Other chemotherapy including azacytidine, gemcitabine, cyclophosphamide and etoposide were also found to increase CRT level on the cell surface by a similar apoptosis-related mechanism [46, 47]. In addition, when macrophages encounter aged or dysfunctional cells, CRT is secreted from macrophages and binds to a family of asialoglycans on the surface of the target cells to label them for subsequent phagocytosis. Induction of the asialoglycan epitope on cancer cells by the treatment of neuraminidase, a glycoside hydrolase enzyme, was demonstrated to dramatically increase macrophage-secreted CRT binding and phagocytosis of cancer cells [48]. The combination of MAG and neuraminidase is under active investigation in our group.

The traditional classification of macrophages is based on the expression pattern of a set of markers which include transmembrane glycoproteins, scavenger receptors, enzymes, growth factors, hormones, cytokines, and cytokine receptors with diverse functions. However, macrophages are notorious for their “phenotypic plasticity”, that is, the ability to adapt marker expression to the stimuli in the microenvironment. Furthermore, both M1-like and M2-like macrophages are tumor cell phagocytes, indicating that a separate set of markers exist to distinguish phagocytic macrophages from the non-phagocytic ones. Identification of such markers will link phagocytic activities to a macrophage subpopulation, provide more in-depth information on macrophage plasticity and facilitate discovery of new potential targets for manipulating macrophage phagocytosis. Here, we identified CD38, CD209, CD163 and CD206 as potential markers for ES-phagocytic macrophages, as the phagocytic macrophages express significantly higher levels of these surface proteins (Fig. 5). Interestingly, among these proteins, CD38 is an M1-like macrophage marker while the other three are markers of M2-like macrophages. CD38 is a cell surface glycoprotein and extracellular enzyme that functions as a NAD + glycohydrolase and ADPR cyclase. CD38 plays a crucial role in macrophage proliferation and polarization; knocking out or blocking CD38 inhibited LPS induced M1 polarization of macrophages [49]. C-type lectin CD209/DC-SIGN is a cell adhesion and pathogen recognition receptor. Together with scavenger receptors CD163 (hemoglobin-haptoglobin SCR) and CD206 (mannose receptor C type 1), these three are highly expressed in M2-like macrophages and widely accepted as markers for M2 polarized macrophages [50]. In a previous report, CD14, CD206, CD163 and CD209 were identified as surface markers for *E.coli* phagocytic macrophages [15]. In our study, convincing evidence was shown that CD38 but not CD14 is a marker for ES-phagocytic macrophage (Fig. 5). While the discrepancy in these results might be due to differences in techniques (metal-based mass cytometry versus fluorescent-based flow cytometry), it also suggests that phagocytic macrophages can be phenotypically heterogeneous depending on the targets of phagocytosis (bacteria versus cancer cell). Indeed, it was shown that macrophages recognize highly heterogeneous targets via a vast repertoire of pattern recognition receptors [51]. Another support to this notion is our observation that CD163 and CD206 seemed to be markers for macrophages phagocytose DOX- and/or MAG-treated but not non-treated ES cells (Fig. 5B).

Although we demonstrated significant anti-tumor efficacy of MAG and DOX combinatorial therapy against ES in vivo, there were several small tumors that persisted in the combination treatment group. Results from the RNAseq and CIBERSORTx analyses of the tumors showed that MAG increased infiltration of monocytes and all subtypes of macrophages (Fig. 7C). Consistently, we found that MAG led to an increase in expression of genes in chemotaxis and leukocyte migration (Fig. 7D). Previous studies on the mechanism of action of other CD47 blockade such as CC-90002 and SRF231 also identified increased tumor macrophage infiltration and induction of macrophage cytokines [52]. Interestingly, we found that combination of MAG and DOX only induced an increase in tumor infiltration of M2 macrophages (Fig. 7C). We looked closely to the genes differentially expressed in the D&M versus DOX treated tumors and found that among the top down-regulated are genes that promote M1 or inhibit M2 macrophage recruitment and polarization including Pvalb, Pck1, and the top up-regulated genes include Gpnmb which promotes M2 macrophage polarization (Fig. 7B). However, it is well-known that M2 macrophages are highly proficient in phagocytosing tumor cells as we also demonstrated in the current study (Figs. 2, 4 and 6). Intriguingly, we found that Cd209f and Cd209g, the homologs to human CD209 that we identified as a potential marker for phagocytic macrophages (Fig. 5), was downregulated in the tumors treated with D&M (Fig. 7B and Supplemental Fig. 2E). It is tempting to speculate that the decrease in phagocytic activity of infiltrating M2 macrophages is a potential mechanism of resistance to the D&M combinatorial therapy. However, the number of differentially expressed genes that we identified in the current study is too small to allow us to make a statistically significant conclusion. Deeper and more insightful analyses such as spatial single cell RNAseq are warranted to uncover the mechanism of resistance to the combinatorial therapy and will be the focus of future investigation. Nevertheless, it will be important to identify additional markers for macrophage populations responsible for tumor cell clearance and investigate approaches to enhancing the macrophage phagocytic activity specifically associated with these markers as a rational combination with CD47 blockade therapy.

One limitation in the current study is the lack of an immune competent animal model. It is unfortunate that no syngeneic animal models are available for ES; this pediatric bone cancer only develops in humans. A potential approach to overcoming this limitation is utilizing humanized mouse model. However, establishing human immune system in mice takes at least 12–16 weeks at which point the mice already reach adulthood and therefore are not ideal to study pediatric cancers. Furthermore, humanized mouse models have their own limitations due to high cost, short animal lifespan and unable to fully recapitulate human immune system [53]. A challenge in translating MAG into the clinic is its side effects, especially anemia due to CD47 expression on erythrocytes. Fortunately, anemia can be managed by using a priming dose of MAG at 1 mg/kg followed by maintenance doses ranging from 3 to 45 mg/kg [54]. Alternatively, MAG can be replaced by the SIRPα/Fc fusion protein or next generation anti-CD47 antibodies that have minimal binding to erythrocytes [55].

## Conclusion

In summary, in the current study, we provide preclinical evidence that CD47 blockade combined with chemotherapy significantly enhanced macrophage phagocytosis of ES cells in vitro and significantly decreased ES xenograft tumor growth and metastasis in vivo. Our data highlight a highly translatable alternative approach in ES innate based immunotherapy resulting in a re-education of macrophages as another arm of the immune system that can engage in anti-tumor activity thereby potentially improving outcomes in patients with poor risk ES.

### Supplementary Information

Additional file 1.

Additional file 2.

Additional file 3: Supplemental Figure 1. Schematic representation of strategies to enhance macrophage phagocytosis of tumor cells. Macrophage phagocytosis is regulated by the balance between the anti-phagocytic “don’t eat me” signal and the pro-phagocytic “eat me” signal. When tumor cells express high “don’t eat me” signal (CD47/SIRPα), blocking the “don’t eat me” signal by CD47 blockade (MAG) enhances phagocytosis. When tumor cells express low or no “eat me” signal (CD91/CRT), induction of “eat me” signal enhances phagocytosis. Chemotherapy drugs such as Doxorubicin (Dox) was known to enhance translocation of intracellular CRT to the surface of cancer cells during the process of apoptosis. When tumor cells express high “don’t eat me” signal and low or no “eat me” signal at the same time, blocking the “don’t eat me” signal and inducing the “eat me” signal simultaneously is required to boost macrophage phagocytosis.

Additional file 4: Supplemental Figure 2. Volcano plots showing differentially expressed genes (DEGs) between groups. A threshold for DEGs between two groups was set to an absolute value of fold change (FC) ≥ 1.5 and a false discovery rate of ≤ 0.10. High means FC greater than 2.5, Low means FC less than -2.5 and Mid means FC in between 2.5 and -2.5. A, Human DEGs comparing D&M vs MAG. B, Human DEGs comparing CON vs D&M. C, Human DEGs comparing DOX vs D&M. D, Human DEGs comparing DOX vs MAG. E, Mouse DEGs comparing D&M vs MAG. F, Mouse DEGs comparing CON vs MAG. G, Mouse DEGs comparing DOX vs MAG.

Additional file 5: Supplemental Figure 3. Pathway and gene ontology enrichment analyses of DEGs via DAVID. Human and mouse DEG sets were submitted to the DAVID (david.abcc.ncifcrf.gov) for enrichment analysis with the Functional Annotation Tool, where OFFICIAL_GENE_SYMBOL was selected and the whole genome of *Homo sapiens* and *Mus musculus* were used as the background genes, respectively. A, Human downregulated DEGs comparing CON vs MAG. B, Human upregulated DEGs comparing CON vs D&M. C, Human downregulated DEGs comparing D&M vs MAG. D, Human downregulated DEGs comparing CON vs D&M. E, Human upregulated DEGs comparing D&M vs MAG. F, Mouse upregulated DEGs comparing CON vs MAG. G, Mouse upregulated DEGs comparing D&M vs MAG. H, Mouse downregulated DEGs comparing DOX vs MAG. I, Mouse downregulated DEGs comparing CON vs MAG. J, Mouse downregulated DEGs comparing D&M vs MAG.

## Data Availability

Raw RNA-Seq data is available in GEO (GSE244548). Other data and material are available from the corresponding author on reasonable request.

## Abbreviations

ANOVA: Analysis of variance
CRISPR: Clustered regulatory interspaced short palindromic repeats
csCRT: Cell surface calreticulin
D&M: DOX and MAG
DOX: Doxorubicin
ES: Ewing sarcoma
KO: Knockout
MAG: Magrolimab
PDX: Patient derived xenograft
TAM: Tumor associated macrophage
TME: Tumor microenvironment
