# Supplementary material for: Combinatorial macrophage induced innate immunotherapy against Ewing sarcoma: Turning “Two Keys” simultaneously
Source: J Exp Clin Cancer Res. 2024 Jul 11;43:193. doi: 10.1186/s13046-024-03093-w (PMC11238356; doi:10.1186/s13046-024-03093-w)
Supplement: Supplementary file 3 — Additional file 3: Supplemental Figure 1. Schematic representation of strategies to enhance macrophage phagocytosis of tumor cells. Macrophage phagocytosis is regulated by the balance between the anti-phagocytic “don’t eat me” signal and the pro-phagocytic “eat me” signal. When tumor cells express high “don’t eat me” signal (CD47/SIRPα), blocking the “don’t eat me” signal by CD47 blockade (MAG) enhances phagocytosis. When tumor cells express low or no “eat me” signal (CD91/CRT), induction of “eat me” signal enhances phagocytosis. Chemotherapy drugs such as Doxorubicin (Dox) was known to enhance translocation of intracellular CRT to the surface of cancer cells during the process of apoptosis. When tumor cells express high “don’t eat me” signal and low or no “eat me” signal at the same time, blocking the “don’t eat me” signal and inducing the “eat me” signal simultaneously is required to boost macrophage phagocytosis. [file 13046_2024_3093_MOESM3_ESM.pdf]

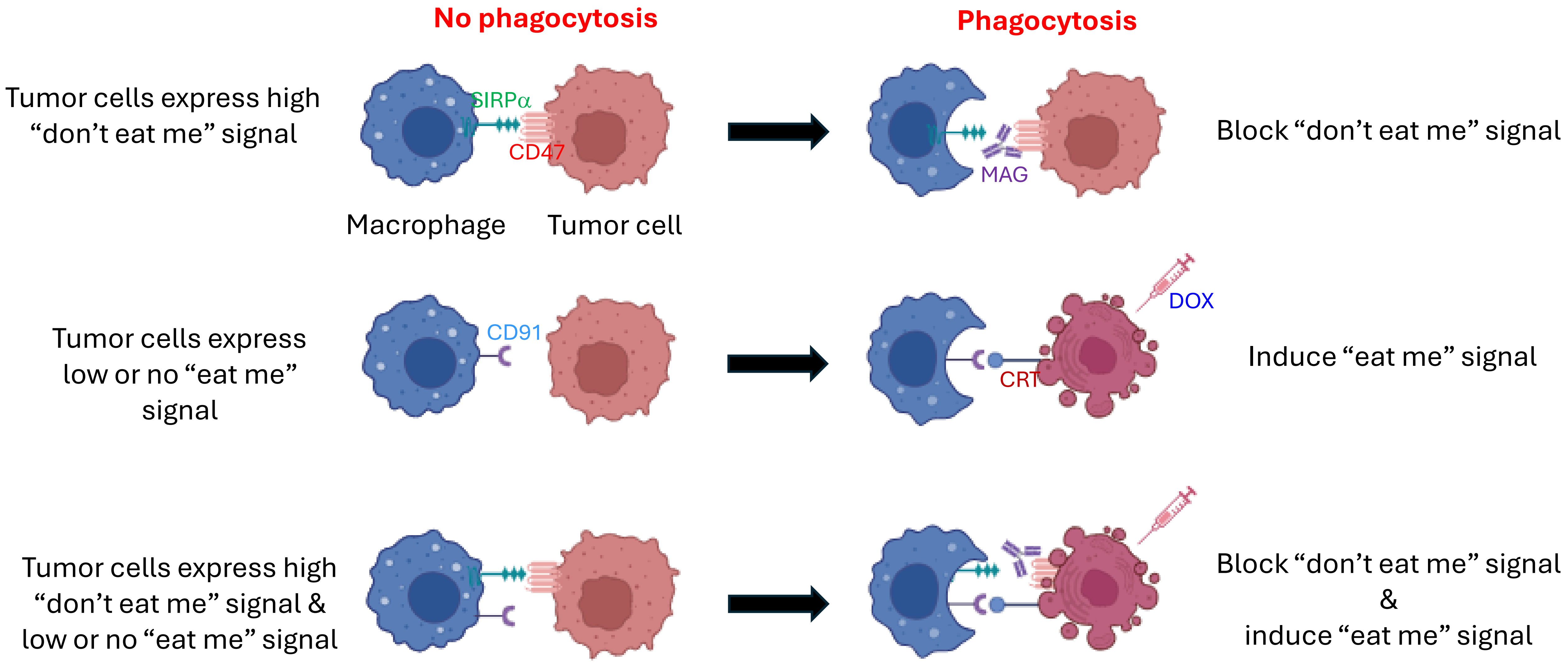

Supplemental Figure 1
