# Supplementary material for: Combinatorial macrophage induced innate immunotherapy against Ewing sarcoma: Turning “Two Keys” simultaneously
Source: J Exp Clin Cancer Res. 2024 Jul 11;43:193. doi: 10.1186/s13046-024-03093-w (PMC11238356; doi:10.1186/s13046-024-03093-w)
Supplement: Supplementary file 4 — Additional file 4: Supplemental Figure 2. Volcano plots showing differentially expressed genes (DEGs) between groups. A threshold for DEGs between two groups was set to an absolute value of fold change (FC) ≥ 1.5 and a false discovery rate of ≤ 0.10. High means FC greater than 2.5, Low means FC less than -2.5 and Mid means FC in between 2.5 and -2.5. A, Human DEGs comparing D&M vs MAG. B, Human DEGs comparing CON vs D&M. C, Human DEGs comparing DOX vs D&M. D, Human DEGs comparing DOX vs MAG. E, Mouse DEGs comparing D&M vs MAG. F, Mouse DEGs comparing CON vs MAG. G, Mouse DEGs comparing DOX vs MAG. [file 13046_2024_3093_MOESM4_ESM.pdf]

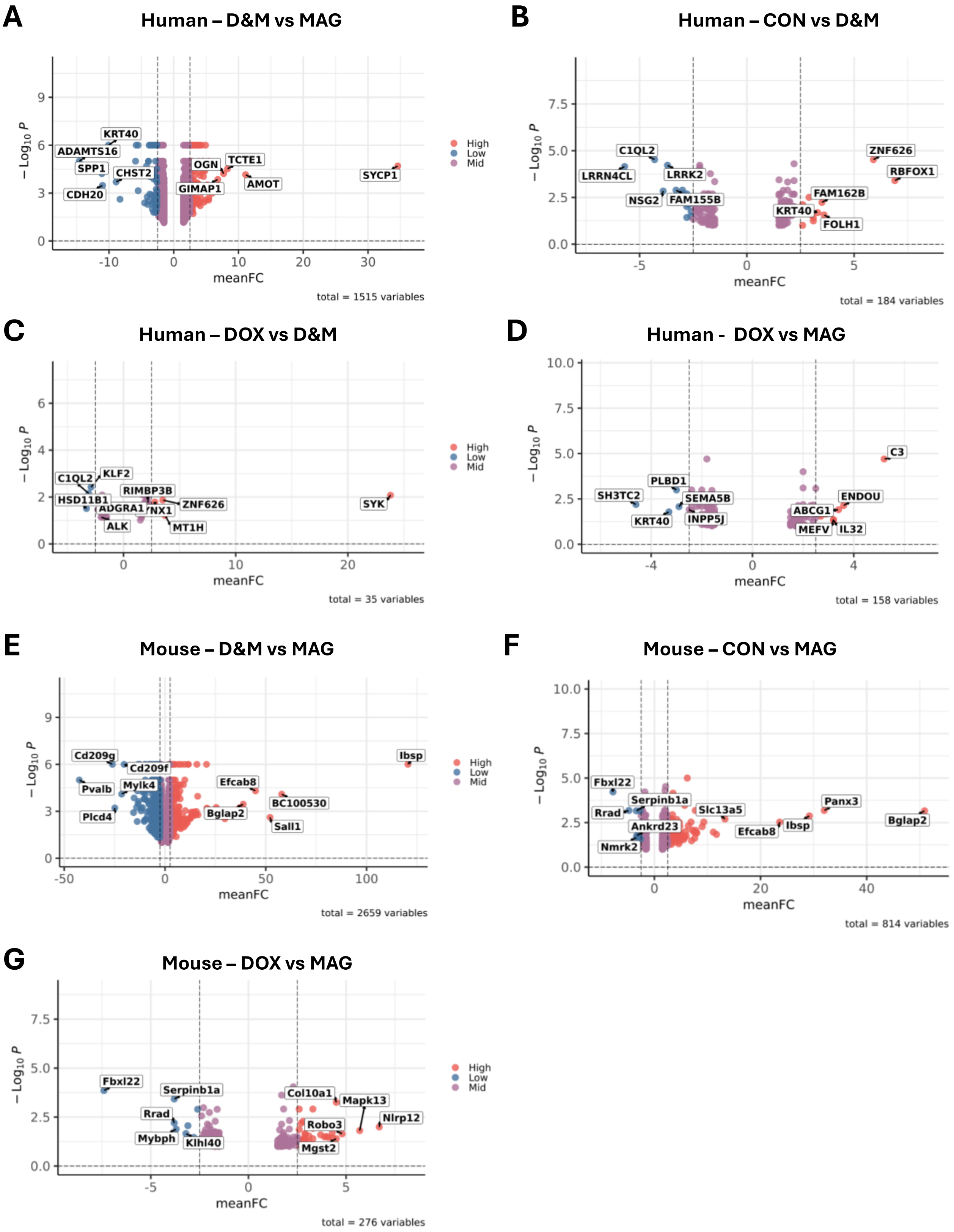
A
Human – D&M vs MAG
KRT40
ADAMTS16
SPP1
CHST2
CDH20
OGN
TCTE1
GIMAP1
AMOT
SYCP1
meanFC
total = 1515 variables
B
Human – CON vs D&M
C1QL2
LRRN4CL
LRRK2
NSG2
FAM155B
ZNF626
RBFOX1
FAM162B
KRT40
FOLH1
meanFC
total = 184 variables
C
Human – DOX vs D&M
C1QL2
KLF2
HSD11B1
RIMBP3B
ADGRA1
ZNF626
ALK
MT1H
SYK
meanFC
total = 35 variables
D
Human - DOX vs MAG
PLBD1
SH3TC2
SEMA5B
KRT40
INPP5J
C3
ENDOU
ABCG1
MEFV
IL32
meanFC
total = 158 variables
E
Mouse – D&M vs MAG
Cd209g
Cd209f
Pvalb
Mylk4
Plcd4
Efcab8
BC100530
Bglap2
Sall1
Ibsp
meanFC
total = 2659 variables
F
Mouse – CON vs MAG
Fbxl22
Serpinb1a
Rrad
Slc13a5
Ankrd23
Nmrk2
Efcab8
Ibsp
Panx3
Bglap2
meanFC
total = 814 variables
G
Mouse – DOX vs MAG
Fbxl22
Serpinb1a
Rrad
Mybph
Klhl40
Col10a1
Mapk13
Nlrp12
Robo3
Mgst2
meanFC
total = 276 variables
High
Low
Mid
