# Supplementary figures and images for: Combinatorial macrophage induced innate immunotherapy against Ewing sarcoma: Turning “Two Keys” simultaneously
Source: J Exp Clin Cancer Res. 2024 Jul 11;43:193. doi: 10.1186/s13046-024-03093-w (PMC11238356; doi:10.1186/s13046-024-03093-w)

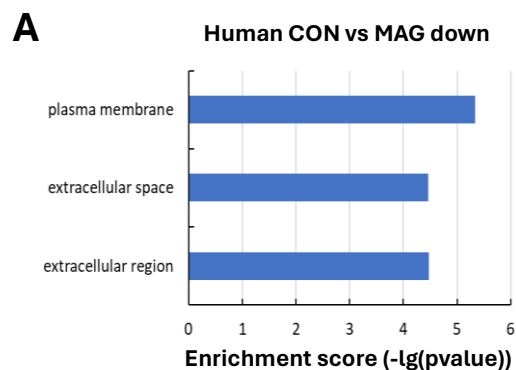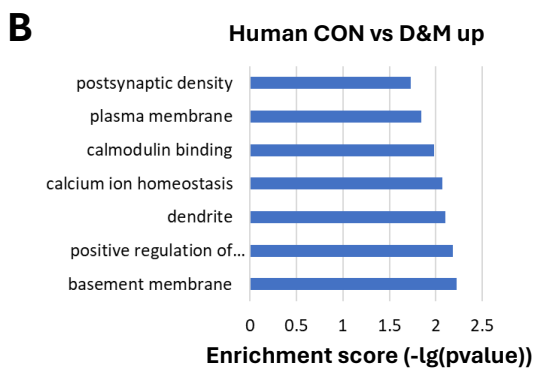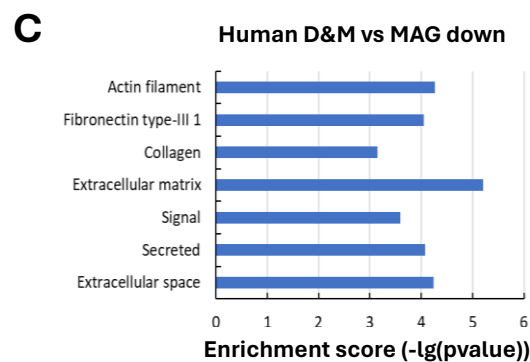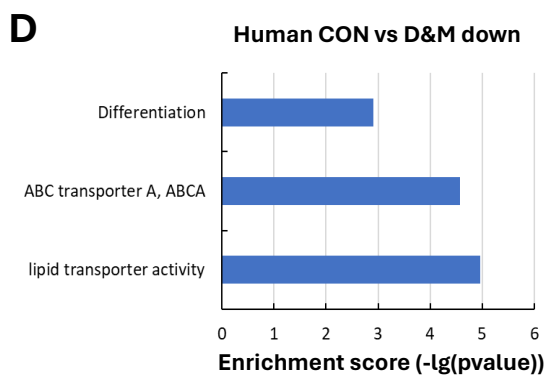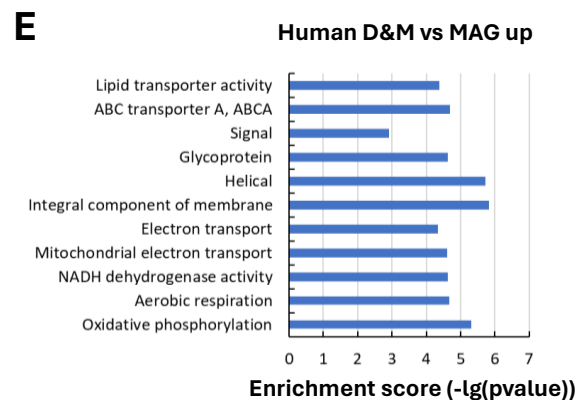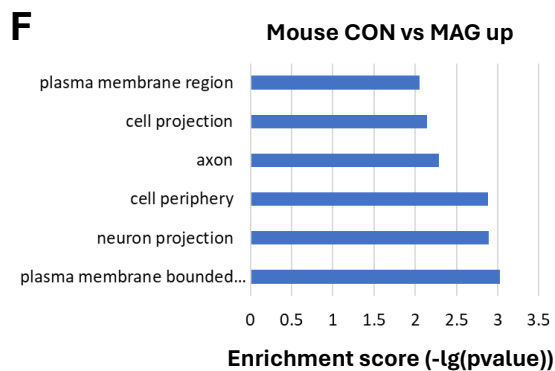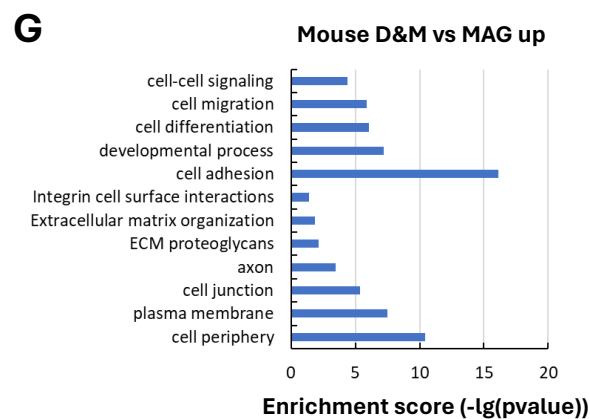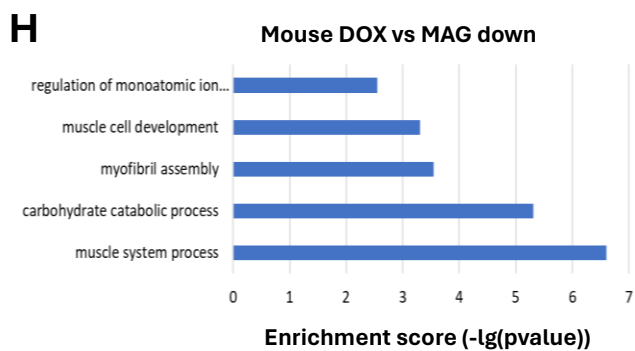

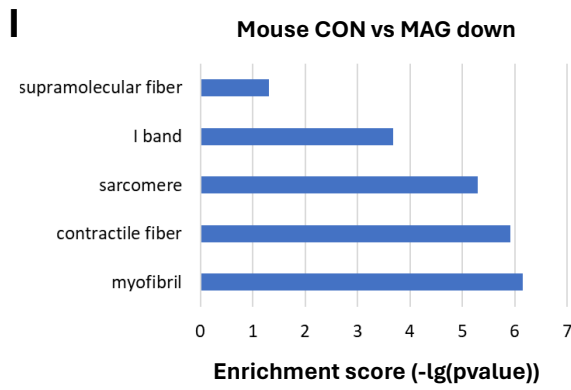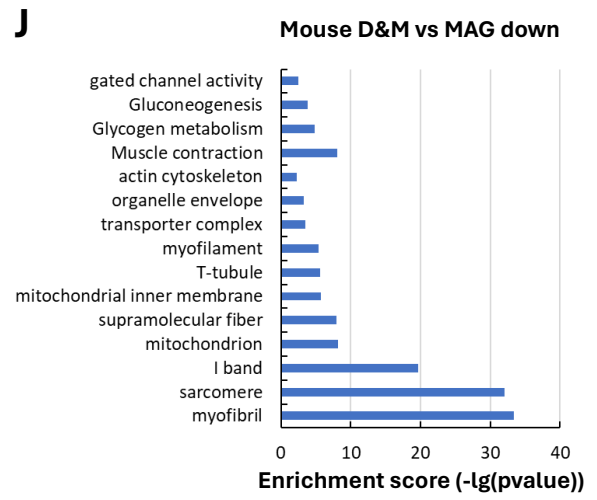

**Supplemental Figure 3**

Supplement: Supplementary file 5 — Additional file 5: Supplemental Figure 3. Pathway and gene ontology enrichment analyses of DEGs via DAVID. Human and mouse DEG sets were submitted to the DAVID (david.abcc.ncifcrf.gov) for enrichment analysis with the Functional Annotation Tool, where OFFICIAL_GENE_SYMBOL was selected and the whole genome of Homo sapiens and Mus musculus were used as the background genes, respectively. A, Human downregulated DEGs comparing CON vs MAG. B, Human upregulated DEGs comparing CON vs D&M. C, Human downregulated DEGs comparing D&M vs MAG. D, Human downregulated DEGs comparing CON vs D&M. E, Human upregulated DEGs comparing D&M vs MAG. F, Mouse upregulated DEGs comparing CON vs MAG. G, Mouse upregulated DEGs comparing D&M vs MAG. H, Mouse downregulated DEGs comparing DOX vs MAG. I, Mouse downregulated DEGs comparing CON vs MAG. J, Mouse downregulated DEGs comparing D&M vs MAG. [file 13046_2024_3093_MOESM5_ESM.pdf]
